# Introduction of High-Sensitivity Troponin Assay to Rural Tertiary Care Medical Center: Impact on Subsequent Cardiac Diagnostic Testing

**DOI:** 10.3390/diagnostics16111738

**Published:** 2026-06-05

**Authors:** Peter M. Leonard, Alexandra Ortengren, Adam Z. Spitz, Roshni Kalkur, Pablo Martinez-Camblor, Jose Mercado, Kerrilynn Hennessey, Cynthia C. Taub

**Affiliations:** 1 Department of Internal Medicine, Dartmouth Hitchcock Medical Center, Lebanon, NH 03766, USA; peter.m.leonard@hitchcock.org (P.M.L.);; 2 Department of Cardiology, Heart and Vascular Center, Dartmouth Hitchcock Medical Center, Lebanon, NH 03766, USA; robertson.alliea@gmail.com; 3 Department of Anesthesiology, Dartmouth Hitchcock Medical Center, Lebanon, NH 03766, USA; pablo.martinez-camblor@hitchcock.org (P.M.-C.);; 4 Department of Medicine, SUNY Upstate University Hospital, Syracuse, NY 13210, USA

**Keywords:** high-sensitivity cardiac troponin, cascade testing, rural hospitals, stress testing utilization, troponin testing, chest pain

## Abstract

High-sensitivity cardiac troponin (hs-cTn) assay offers improved diagnostic accuracy for the detection of myocardial infarction but has seen slow adoption in the United States, particularly in rural settings. Limited research has explored the impact of hs-cTn implementation on downstream testing, in rural hospitals where diagnostic delays are common. This study evaluates the effects of transitioning from standard assay to hs-cTn in a rural tertiary care medical center. We conducted a retrospective analysis of de-identified data from four weeks before and after the implementation of an hs-cTn assay. We assessed the presence of downstream cardiovascular testing and consults among emergency department and inpatient chest pain encounters. Lengths of stay were evaluated. In total, 1664 pre- and 1479 post-implementation admissions were evaluated. Demographic characteristics and comorbidities were similar during the study period. Chest pain was reported more frequently during the post-hs-cTn implementation period as the chief complaint (21.4% vs. 28.7%; *p* = 0.0036). There was a statistically significant decrease in the number of downstream stress tests performed in the second period (aOR 0.55, 95% CI: 0.31–0.98). The adjusted odds ratio (aOR) for cardiac catheterization trended toward a non-significant decrease (aOR 0.57, 95% CI: 0.40–1.11). Lengths of stay were similar between groups. In a retrospective analysis of patients who underwent cardiac troponin assays, we found that the implementation of hs-cTn was associated with a decrease in downstream stress testing without a significant increase in the number of cardiology consultations, coronary angiographies, or coronary computed tomography scans.

## 1. Introduction

High-sensitivity cardiac troponin assay (hs-cTn) offers increased sensitivity and higher diagnostic accuracy, thereby enabling more rapid detection and treatment for acute myocardial infarction, as compared with traditional cardiac troponin assay.

Cardiac troponin serves as a primary marker of myocardial damage, which has broad clinical utility beyond acute coronary syndromes. Elevated troponin levels are associated with a variety of acute cardiac conditions including pulmonary embolism, acute decompensated heart failure, and cardiac arrythmias. Further, elevations can be seen in the context of non-cardiac critical illness such as sepsis, respiratory failure, and acute kidney injury [1,2]. Beyond acute illness, the baseline cardiac troponin level has been observed to correlate with subclinical myocardial dysfunction and may serve as a prognostic marker for adverse cardiovascular outcomes in asymptomatic populations [3,4,5].

Despite the superiority of this assay, its implementation has been slow. Following the initial development in 2010, the use of hs-cTn had been primarily limited to countries outside the United States (US) until the assay gained Food and Drug Administration approval in 2017. Since approval, the rollout of the hs-cTn assay through the US has similarly been slow. In a 2023 study, the implementation of hs-cTn assays among participating hospitals had increased from 3.3% to only 32.6%, from 2019 to late 2021, with the majority of US hospitals continuing to use the less sensitive assay [6]. Uptake has been even more limited among rural hospitals, as urban hs-cTn implementation outpaced that of rural medical centers [6,7]. Rural hospitals face greater delays in the diagnosis and treatment of myocardial infarction due to greater transport times to percutaneous coronary intervention (PCI)-capable facilities; therefore the uptake of an assay with improved sensitivity is paramount [8].

One potential contributor to the delay in the uptake of hs-cTn may be concern regarding the safety and operational impact of uptake in a rural environment. An analysis of European centers showed an increase in coronary angiography after introducing hs-cTn [9].

Previous evaluations of cascade testing in the emergency department demonstrated that the transition to an hs-cTn assay led to a 3% net increase in cascade events, though these were limited to electrocardiograms (ECGs) and multiple troponin assays [10]. Patients ultimately received fewer computed tomography (CT) scans, stress tests, and cardiac catheterizations. Patients were also less likely to be hospitalized for chest pain-related complaints and had shorter lengths of stay [10]. A similar study found that a transition to hs-cTn assay led to increased coronary angiography utilization and a reduction in length of stay, echocardiography, and stress testing [11]. Each of these studies were conducted primarily in suburban or urban hospital centers with shorter transport times to PCI-capable facilities. There has been limited research in the US on the transition from traditional assays to hs-cTn and how cascades of downstream services are affected in rural environments [6]. Prior implementation studies have not detailed educational frameworks beyond basic protocol dissemination, particularly in rural settings where clinician familiarity with hs-cTn interpretation varies widely [12]. The purpose of this study is to evaluate the operational impact of a comprehensive system-wide educational program and guided transition to 5th-generation high-sensitivity troponin T in a rural tertiary care medical center.

## 2. Materials and Methods

### 2.1. Design and Data Collection

In this single-center retrospective study, we assessed a de-identified dataset containing all encounters from the emergency department and inpatient services four weeks pre- and four weeks post-introduction of a high-sensitivity troponin assay. Data was collected from August 2022 to October 2022. Encounters were included regardless of indication for troponin testing, while encounters associated with patients below 18 years old were excluded. The binary presence or absence of orders for ECGs, coronary computed tomography angiograms (coronary CTA), stress testing (nuclear or echocardiographic stress test), cardiac catheterizations, and cardiovascular consult requests was quantified as outcomes. Only orders for downstream testing placed or obtained during the index encounter were included in the analysis.

Demographical and clinical variables including age, sex, race, and comorbid conditions (chronic kidney disease, congestive heart failure, coronary artery disease, hypertension, hyperlipidemia, or diabetes mellitus) were collected. Chief complaints were recorded. Lengths of stay were tabulated.

### 2.2. Microlearning and System-Wide Education

As part of an implementation strategy for the hs-cTn assay, a microlearning-based program was developed and deployed at our rural academic medical center. The approach included short, focused educational videos which explained the differences between hs-cTn and traditional troponin assays and the institutional algorithm and allowed providers to work through case examples. To complement the videos, interactive decision support tools were also made available to reinforce key concepts and promote adherence to the algorithm. Content was periodically updated.

### 2.3. Statistical Analysis

Analysis was performed at the admission level. We assumed independence between the admission units even when they are from the same patient. We used standard descriptive analysis to summarize variables. Continuous variables were described by the mean ± standard deviation (SD) when symmetric or the first, second (median), and third quantiles when asymmetric. The Shapiro–Wilk test was used to test the normality of the data. Absolute and relative counts described categorical variables. For group comparisons, Welch, Kruskal–Wallis or Chi-2 tests were used as appropriate. Odds ratios derived from uni- and multivariable logistic regression models were used to summarize the raw and adjusted relationships between the main binary outcomes and the exposure variable (period). Considered covariates included: chest pain as the chief complaint, age (yrs), sex (Man/Woman), heart failure, chronic kidney disease, coronary artery disease, hypertension, hyperlipidemia, and diabetes. Given the asymmetry of the lengths of stay, uni- and multivariable quartile regression for the median was used. The resulting coefficients (Beta) were used to measure the unadjusted and adjusted (same pool of covariates was considered) association between this outcome and exposure. Odds ratios with 95% confidence intervals derived from logistic regressions are reported for binary outcomes, while coefficients and 95% confidence intervals associated with quantile regressions for the median are reported for continuous outcomes. Analyses were performed using a complete-case approach. A *p* value < 0.05 was considered statistically significant. All analyses were performed in the statistical environment R 4.5.3 (www.r-project.org).

## 3. Results

A total of 1526 and 1347 patients were evaluated in the pre- and post-hs-cTn groups, for a total of 1664 and 1479 admissions, respectively. Analysis was performed at the admission level; therefore, there were 1664 and 1479 admissions in each group.

Baseline characteristics (Table 1) were similar between groups. Age ranged from 19 to 104.8 years, with an average of 66.9 ± 17.6. A total of 49.7% of patients were male, and 97.3% self-reported as being of white race. The presence of coronary artery disease (CAD), chronic kidney disease (CKD), hypertension (HTN), hyperlipidemia (HLD), and diabetes was similar between groups.

**Table 1:** Baseline characteristics before (Period 1) and after (Period 2) implementation of high-sensitivity cardiac troponin.

Characteristic	Period 1 (*n* = 1664)	Period 2 (*n* = 1479)	*p* Value
Age, mean (SD), y ^a^	67 (17)	66 (17)	0.34
Male sex, No. (%)	834 (50.1)	727 (49.2)	0.61
White race, No. (%)	1625 (97.7)	1434 (97.0)	0.27
Heart failure, No. (%)	661 (39.7)	598 (40.4)	0.71
Chronic kidney disease, No. (%)	224 (13.5)	198 (13.4)	0.99
Coronary artery disease, No. (%)	416 (25.0)	360 (24.3)	0.70
Hypertension, No. (%)	691 (41.5)	639 (43.2)	0.36
Hyperlipidemia, No. (%)	609 (36.6)	552 (37.3)	0.70
Diabetes, No. (%)	294 (17.7)	261 (17.7)	>0.99

Abbreviation: SD, standard deviation. ^a^ Plus–minus values are presented as mean (SD).

Chest pain was reported more frequently during the second period (Table 2), both as the chief complaint (*p* value = 0.0036) and as a reason for presentation (*p* value = 0.0029). Chest pain as the chief complaint refers to the principal symptom identified at triage, whereas chest pain as a reason for presentation refers to the presence of chest pain among multiple presenting symptoms.

**Table 2:** Presence of chest pain by time period.

Variable	Period 1, No./Total (%)	Period 2, No./Total (%)	*p* Value
Chest pain as a reason for presentation	172/649 (26.5)	211/614 (34.4)	0.003
Chest pain as the chief complaint	139/649 (21.4)	176/614 (28.7)	0.004
Chest pain revisit ^a^	13/1664 (0.8)	19/1479 (1.3)	0.22

^a^ Revisit refers to a repeat emergency department visit for chest pain after an initial encounter. Data are reported on a complete-case basis.

### 3.1. Downstream Cardiovascular Testing

We analyzed both unadjusted and adjusted models, with the latter controlling for chest pain, age, sex, and comorbidities, for downstream cardiovascular imaging utilizations. Across all five utilization outcomes (cardiac catheterization, cardiology consult request, stress test, coronary CTA and ECG), the results were similar between groups in both models (Table 3).

**Table 3:** Cardiac testing and cardiology consultation utilization before and after implementation of high-sensitivity cardiac troponin.

	Unadjusted			Adjusted		
Testing Modality	OR	95% CI	*p* Value	OR	95% CI	*p* Value
Cardiac catheterization	1.15	0.85–1.57	0.39	0.67	0.40–1.11	0.12
Cardiology consultation	0.80	0.50–1.28	0.35	0.82	0.50–1.32	0.40
Stress test	0.61	0.39–0.96	0.03	0.55	0.31–0.98	0.04
Coronary CTA	2.25	0.20–24.86	0.51	0.89	0.05–17.06	0.94
ECG	1.14	0.86–1.53	0.36	1.00	0.58–1.71	0.99

Abbreviations: CI, confidence interval; CTA, computed tomography angiography; ECG, electrocardiogram; OR, odds ratio.

Adjusted analyses showed non-significant trends toward reduced cardiology consultation (aOR 0.82, 95% CI 0.50 to 1.32) and cardiac catheterization (aOR 0.67, 95% CI 0.40 to 1.11); however the unadjusted estimate for cardiac catheterization (OR 1.15, 95% CI 0.85 to 1.57) was directionally opposite. ECG utilization was unchanged. After the implementation of hs-cTn, there was a statistically significant reduction in downstream stress testing (3.4% (56/1664) and 2.1% (31/1479), in the pre and post periods, respectively), with both unadjusted (OR 0.61, 95% CI 0.39 to 0.96, *p* = 0.0319) and adjusted models (aOR 0.55, 95% CI 0.31 to 0.98, *p* = 0.0432) showing that fewer studies were performed. Coronary CTA was performed in only three encounters across both periods combined. The wide confidence interval for this outcome (OR 2.25, 95% CI 0.20 to 24.86; aOR 0.89, 95% CI 0.05 to 17.06) reflects sparse events, and this estimate should be interpreted with caution.

### 3.2. Lengths of Stay

After adjusting for covariates, the median emergency department (ED) lengths of stay were larger in the second period (Table 4, B = 0.71, 95% CI 0.17 to 1.25, *p* = 0.0099). No significant difference was observed in the hospital lengths of stay between periods in either unadjusted or adjusted analyses.

**Table 4:** Unadjusted and adjusted inpatient and emergency department lengths of stay before and after implementation of high-sensitivity cardiac troponin.

	Unadjusted			Adjusted		
Length of Stay	β	95% CI	*p* Value	β	95% CI	*p* Value
Hospital	−0.28	−4.76 to 4.23	0.90	−3.42	−10.46 to 3.62	0.34
Emergency department	0.25	−0.39 to 0.89	0.44	0.71	0.17–1.25	0.01

Abbreviation: CI, confidence interval.

## 4. Discussion

High-sensitivity cardiac troponin assays are recommended by the American College of Cardiology (ACC) and American Heart Association (AHA) for the early detection and risk stratification of myocardial injury. Yet adoption in rural centers remains limited, in part due to concerns of potential increases in downstream testing in resource-constrained environments.

The findings of our study were 2-fold.

- After the implementation of a high-sensitivity troponin assay, we observed no significant increase in downstream testing and in fact saw a significant reduction in non-invasive stress testing.
- A microlearning-based educational strategy was implemented as part of a broader system-wide transition; however, the independent effect of this intervention on clinician behavior or protocol adherence was not directly measured.

We analyzed the utilization of five resources before and after the implementation of high-sensitivity troponin testing. Although there was concern that cardiology consultation would increase due to more positive troponins being detected after high-sensitivity assay adoption, no increase in consultation was observed, which is consistent with prior multicenter urban studies [10]. We also observed a significant reduction in functional stress test utilization in the post-implementation period, which is also consistent with prior urban and rural studies [10,11,13]. This reduction is likely reflective of increased confidence in the predictive value of a negative high-sensitivity troponin assay. This decrease is particularly relevant in rural settings, where access to advanced imaging modalities is often limited, and unnecessary testing may contribute to delays in definitive care. Newer-generation assays may further amplify this effect. A recent multicenter cohort study evaluating a sixth-generation hs-cTn assay identified nearly twice as many patients as low-risk at presentation compared to the fifth-generation assay used in our institution, with similar negative predictive value [14].

Several prior studies, including most notably a rural multicenter study, have shown an increase in coronary angiography after the implementation of a high-sensitivity troponin assay [9,11,15]. We observed no such increase in invasive testing in our post-implementation period. This finding is especially reassuring in low-resource hospitals, where unnecessary transfers for invasive testing may impose logistical and financial burdens on both patients and healthcare systems. The absence of increased angiography in our study suggests that structured education and strategic implementation can mitigate the overutilization of invasive testing. Additionally, prior multicenter urban studies revealed an increase in upfront testing such as ECGs after high-sensitivity troponin implementation; however, our study identified no change in ECG utilization [10]. Many troponin elevations identified by high-sensitivity assays represent Type 2 myocardial infarction or non-ischemic myocardial injury, in which the role of invasive angiography is less defined [16]. This may account for the stable rates of coronary angiography in our cohort despite the increased detection of troponin.

While there is ample evidence of the successful implementation of high-sensitivity troponin assays in urban centers, the literature on rural implementation, where resources are scarcer, is lacking [10,17,18,19]. The implementation studies that exist for rural centers unfortunately have shown an increase in the utilization of invasive coronary angiography after high-sensitivity implementation [11].

Our study highlights the operational feasibility of implementing high-sensitivity troponin assays in a rural academic medical center, with reduced stress test utilization and no increase in invasive downstream testing. Implementation was accompanied by a microlearning-based educational program using brief videos and interactive tools. Because we did not measure clinician engagement with the materials, adherence to the diagnostic algorithm, or ordering behavior fidelity, the independent contribution of microlearning to the observed changes cannot be determined and remains hypothesis-generating. Microlearning may nonetheless be a feasible educational format in rural settings, where clinicians have limited time for traditional didactic sessions, and warrants prospective evaluation [20,21].

Interactive tools providing immediate feedback allow clinicians to apply their knowledge in practice-based scenarios. Educational strategies such as microlearning may be especially well-suited for rural centers, where providers often manage diverse clinical responsibilities, limiting the time available for lengthy didactic sessions. Furthermore, compared to traditional education strategies, the nature of microlearning also allows for more regular updates reflecting the latest guidelines.

While we observed reductions in downstream stress testing following hs-cTn implementation, these findings likely reflect a combination of factors, including assay performance characteristics, institutional protocols, and accompanying education efforts. The specific contribution of the microlearning intervention remains hypothesis-generating and warrants prospective evaluation.

Like other institutions, our implementation strategy involved extensive multidisciplinary education involving emergency, cardiovascular, and hospital medicine as well as clinical pathology. However, an additional aspect of our implementation was duplicate troponin testing with both the low- and high-sensitivity troponin assays being used for the first two weeks after implementation. This allowed clinicians to directly compare the values of both tests, contextualize the results, and better integrate the high-sensitivity assay into their practice. While initial duplicate testing does imply a significant upfront cost, it may ultimately be outweighed by the benefit of the more judicious utilization of higher-cost and more invasive testing downstream.

This study should be evaluated in light of a number of limitations and caveats. One limitation of this study is the brief observation window of four weeks before and after implementation. This short interval limits causal interference about the effect of hs-cTn implementation itself and increases the risk of temporal confounding from seasonal admission patterns, concurrent staffing or workflow changes, and secular trends in cardiac testing. The findings should be interpreted as preliminary signals warranting confirmation in studies with longer observation windows. Furthermore, this is a retrospective review of a single-center experience. It is notable that only 28.7% of patients undergoing high-sensitivity troponin testing presented with a chief complaint of chest pain, which may have contributed to our lower rates of downstream testing. This implies that clinical history-taking and physical examinations to assess chest pain continue to play an important role in clinical decision-making in our medical center.

Although our institution is rural, it is a tertiary academic medical center with 24/7 access to emergency percutaneous coronary intervention and cardiac surgery. Additionally, coronary CTA is not routinely performed in the inpatient or emergency department settings at this institution. Nuclear stress testing is not available on weekends. Therefore, our results may not directly translate to the experience of rural community or critical access hospitals.

In addition, we did not measure implementation outcomes such as clinician engagement with educational materials, adherence to diagnostic algorithms, or ordering behavior fidelity. As such, we cannot determine the extent to which the observed changes were mediated by the microlearning-based educational strategy versus other components of the implementation.

## 5. Conclusions

After the introduction of a high-sensitivity troponin assay at a rural tertiary care medical center using a comprehensive educational approach, there was no statistically significant increase in cascade testing or cardiovascular consults. There was a modest decrease in stress testing. These results are consistent with hs-cTn supporting the more selective use of downstream non-invasive testing, although diagnostic confidence and clinician ordering behavior were not directly assessed. A structured educational strategy accompanied implementation; however, its independent effect on clinician behavior and workflow adoption was not directly measured.

## Data Availability

The original contributions presented in this study are included in the article. Further inquiries can be directed to the corresponding author.

## Abbreviations

The following abbreviations are used in this manuscript:

Hs-cTn	High-sensitivity cardiac troponin
OR	Odds ratio
US	United States
PCI	Percutaneous coronary intervention
ECG	Electrocardiogram
CT	Computed tomography
Coronary CTA	Coronary computed tomography angiogram
SD	Standard deviation
CAD	Coronary artery disease
CKD	Chronic kidney disease
HTN	Hypertension
HLD	Hyperlipidemia
ED	Emergency department
ACC	American College of Cardiology
AHA	American Heart Association
